# Biomarker Variability, Severity of CORADS and Impact of Psychotropic Medications on Course and Outcome of Neuropsychiatric Sequalae of COVID-19 ICU Patients: A Preliminary Observation from South India

**DOI:** 10.1192/j.eurpsy.2023.1253

**Published:** 2023-07-19

**Authors:** S. Kandrakonda, V. R. Vatte, S. Penubarthi, S. R. Karri, A. Kamble, P. Khairkar, D. Pustakaala, A. kanlur, S. K. Y. Anna, S. S. Penugonda, R. Gadde, V. K. Inakollu, R. Soorineedu

**Affiliations:** 1 Psychiatry; 2 Biochemistry, Kamineni Institute of Medical Sciences, Narketpally; 3 Pharmacology, NIMS, Hyderabad; 4 Psychiatry, Sri Venkateshwara Medical College, Tirupati; 5 Psychiatry, Government Medical College, Nizamabad, India

## Abstract

**Introduction:**

There is growing evidence of neuropsychiatric presentations in patients of COVID-19, but literature is scarce on laboratory, clinical and radiological markers as well as impact of psychotropic medications during the course of hospitalization in critically ill patients.

**Objectives:**

The primary outcome measure was variability of clinical biomarkers and CORADS scores with severity of COVID-19 infections and the impact of psychotropic medications like risperidone and aripiprazole.

**Methods:**

We screened 430 ICU patients admitted to our tertiary care hospitals, out of whom 67 were diagnosed positively with definitive neuropsychiatric sequalae and receive psychotropic interventions during their hospital stay. We compared their D-dimer levels, C-reactive proteins, serum ferritin levels, serum procalcitonin and Vitamin D levels and further analyzed CORADS severity score with psychiatric severity and outcome.

**Results:**

The mean age of the patients was 42.38 years, majority (44.8%) of them belonged to 21-34 years with slight (52.2%) male preponderance and none of them were more than 60 years. We observed a 43.3% were having organic mood disorder and 37.3% of individual had significant history of alcohol dependence while hypertension and diabetes mellitus were noted in 34.3% and 29.9% respectively. Only D-dimer levels were found to be significant and positively associated with outcome of psychiatric disorders (p<0.05), accounting for 41% of covariance on linear regression analysis.

**Conclusions:**

Our study has found significant association of elevated levels of D-dimer variability but not the other laboratory biomarkers among various neuropsychiatric comorbid sequalae in ICU admitted COVID 19 patients. This particular observation might have potential for serum D-dimer levels to be possibly used as an early biomarker to screen or suspect for comorbid neuropsychiatric presentations.

**Disclosure of Interest:**

None Declared

